# 151. Gastrointestinal infections among US Veterans in the era of multiplex PCR testing

**DOI:** 10.1093/ofid/ofad500.224

**Published:** 2023-11-27

**Authors:** Maryam Munir, Monique Thorne, George Psevdos

**Affiliations:** Stonybrook University Hospital, Northport, New York; Northport VAMC, Northport, New York; Northport VA Medical Center, Northport, New York

## Abstract

**Background:**

Infectious gastroenteritis remains a common cause of morbidity in the US. While stool culture had been the standard tool for microbiological diagnosis, known for its time consuming and often lower yields, rapid diagnostic tests have now been developed. Individual and multiplex gastrointestinal (GI) polymerase chain reaction (PCR) are commercially available that can provide rapid and accurate results. We reviewed the GI infections in US Veterans (VET) assessing the use of GI PCR

**Methods:**

Retrospective chart review from 10/1/2021 to 3/31/2023 of US VET at Northport VAMC who had positive results on Biofire® GI PCR panel; positive ova and parasites (O+P), positive stool cultures, C-difficile GDH/toxin B assays and *H.pylori* stool antigens

**Results:**

In the study period there were 264 GI PCR and 224 O+P tests. 86 VET had GI infections. GI PCR identified 26 viral infections with norovirus being the most common. 16 coinfections identified. The median age was 58.5 years. 77% White, 19% Black. 81 were men. 78/86 had presented with diarrhea, 31 with abdominal pain/dyspepsia. 30 VET were treated as inpatient. 18/86 had Diabetes, 32, GERD, 11 Asthma, 13 COPD, 45 HTN, 46 HLD, 3 HIV, 20 CAD, 7 with malignancies (carcinoid, MALT lymphoma, Non-Hodgins lymphoma, lung, tonsil, prostate, multiple myeloma). 25 VET had recent exposure to antibiotics. 10 had recent travel. 59 received antibiotic therapy. 25 had prior history of *C. difficile* infection (CDI). Of the 30 positive tests by GI PCR, 19 were clinically treated for CDI: 14 with oral vancomycin 5 with fidaxomicin. Of the 6 campylobacter infections by GI PCR, 4 were confirmed by culture. Of the 2 positive vibrio spp by GI PCR: one vibrio cholera was not confirmed by culture and toxin assay (this patient was not included in the analysis); the other vibrio spp by PCR, culture confirmed *V. parahaemolyticus.* 1 VET with multiple myeloma and CDI died due to multiorgan failure. One dual infection with salmonella and Yersinia by PCR: only salmonella (non typhi) was confirmed by culture

GI Infections

**Figure:**
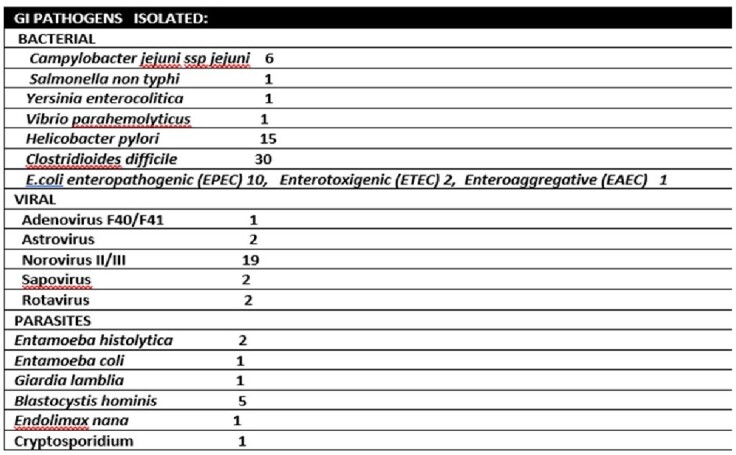

Travel History, Co-Infections

**Figure:**
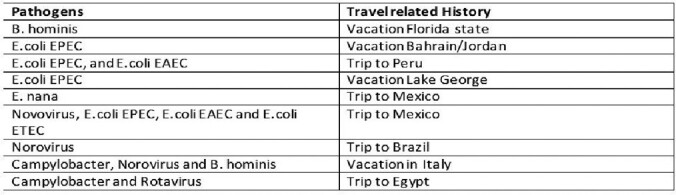

**Conclusion:**

GI PCR can offer a rapid diagnosis for viral etiologies and can be more sensitive as compared to culture for campylobacteriosis. However, vibrio and Yersinia spp results would need to be confirmed by culture. Also diagnosis of CDI would need interpretation of GDH/toxin and clinical judgement

**Disclosures:**

**All Authors**: No reported disclosures

